# Comparison the Efficacy of Fluconazole and Terbinafine in Patients with Moderate to Severe Seborrheic Dermatitis

**DOI:** 10.1155/2014/705402

**Published:** 2014-02-18

**Authors:** Narges Alizadeh, Hamed Monadi Nori, Javad Golchi, Shahriar S. Eshkevari, Ehsan Kazemnejad, Abbas Darjani

**Affiliations:** ^1^Department of Dermatology, Razi Hospital, Guilan University of Medical Sciences, Rasht 41448, Iran; ^2^Department of Preventive and Community Medicine, Guilan University of Medical Sciences, Rasht, Iran

## Abstract

*Background*. Topical agents can be unpleasant due to long-term therapies in patients with moderate to severe seborrheic dermatitis. Systemic antifungal therapy is another alternative in treatment. *Aim*. This study was conducted to compare the efficacy of oral fluconazole and terbinafine in the treatment of moderate to severe seborrheic dermatitis. *Methods*. 64 patients with moderate to severe seborrheic dermatitis (SD) were enrolled in a randomized, parallel-group study. One study group took terbinafine 250 mg daily (*n* = 32) and the other one fluconazole 300 mg (*n* = 32) weekly for four weeks. Seborrheic dermatitis area severity index (SDASI) and the intensity of itching were calculated before, at the end of treatment, and two weeks after treatment. *Results*. Both drugs significantly reduced the severity of seborrheic dermatitis (*P* < 0.001). Multivariate linear regression revealed that efficacy of terbinafine is more than fluconazole (*P* < 0.01, 95% CI (0.63–4.7)). Moreover, each index of SD severity reduced 0.9 times after treatment. (*P* < 0.002, 95% CI (0.8–1.02)). The itching rate significantly diminished (*P* < 0.001); however, there was no difference between these two drugs statistically. *Conclusions*. Both systemic antifungal therapies may reduce the severity index of SD. However, terbinafine showed more reduction in the intensity of the disease. In other words, the more the primary intensity of the disease is, the more its reduction will be. This trial is resgistered with 201102205871N1.

## 1. Introduction

Seborrheic dermatitis (SD) is a common chronic inflammatory skin disorder. It is limited to specific areas of the skin such as the scalp, face, upper trunk, and flexures. It has also been found that there is a relation between overproduction of sebum and *Malassezia* yeast species, which exists naturally in the body [1].

There are different topical remedies to alleviate SD. Regarding disease progression and no response to topical therapy, patients should receive oral treatment. Alternatives are oral antifungal drugswith or without adjuvant therapies. It is the utmost to fulfill the best guideline for severe or persistent disease. Terbinafine is a good choice to decrease severity of SD. However there are alternative drugs to this choice. Recently studies have shown that the fluconazole as a broad spectrum fungistatic drug can be used in treating seborrheic dermatitis. Despite proof of the advantages of fluconazole and terbinafine in the treatment of seborrheic dermatitis, adequate and well-controlled studies concerning the comparison of oral fluconazole and terbinafine are scarce [2–8]. The objective of this study was to compare the efficacy of two antifungal medications in treating moderate to severe seborrheic dermatitis.

## 2. Methods

### 2.1. Study Population

In this open randomized controlled parallel-group clinical study, 64 patients seeking treatment for moderate to severe seborrheic dermatitis in Department of Dermatology, Guilan University of Medical Sciences, Razi Hospital were enrolled. Four patients discontinued the study and sixty patients (28 males and 32 females) participated in this study. They were matched by age and sex in two groups.

This study is launched in October 2008 to March 2011 and took three years and in order to eliminate seasonal impact, the study was conducted only in fall and winter.

Study protocol was approved by the Local Institutional Review Board of Guilan University of Medical Sciences. Before recruitment, written informed consent was obtained. Enrolled patients had no drug history of steroids, antifungal or other topical and systemic therapy for at least two weeks before entering the study (washout time).

All subjects were examined in advance by two calibrated dermatologists with the interexaminer agreement about 90% and the forms were filled out by researchers. Subjects were excluded if they had any of the followings: history of pervious diseases such as chronic renal failure and liver failure, psoriasis, documented human immunodeficiency virus infection and/or sensitivity to fluconazole and terbinafine, breast feeding, and pregnancy. Laboratory tests including CBC, BUN, Cr, AST, ALT, and ALK-Ph were requested for all patients and then the results were controlled and recorded by examiner.

### 2.2. Measuring Seborrheic Dermatitis (Severity Index)

The primary endpoints of study were seborrheic dermatitis area severity index (SDASI) and itching. The scoring system used for assessment of SDASI by Baysal et al. (2004) was applied [9]. “Scalp, face, and chest are examined in the patients, graded for erythema, papule, and scale (0 = absent; 1 = mild; 2 = moderate; 3 = severe). The areas of involvement were measured on a scale of 1 to 5 (1 = less than 10%; 2 = 11–30%; 3 = 31–50%; 4 = 51–70%; 5 = more than 70%)” [9]. Severity of seborrheic dermatitis was divided into three groups based on SDASI scores (mild = 0–7.9, moderate = 8–15.9, and severe > 16).

Itching grading is quoted by Comert (0 = absent; 1 = mild; 2 = moderate; 3 = severe) [2]. The secondary endpoint of study was assessing the side effects of fluconazole and terbinafine.

### 2.3. Sample Size

We considered a one-sided test, 5% significance level with power of 90% and absolute effect size 0.75, SD = 1.2 in the terbinafine and SD = 0.65 in the fluconazole group based on the study of Scaparro et al. and Cömert et al. A sample size of 28 patients per group were determined. Regarding an anticipated dropout rate 10% during three-phase study, 64 patients are enrolled in the study [2, 7].

### 2.4. Treatment

Computerized random allocation was applied with a 1 : 1 allocation using random block size 4. The drugs were sealed in numbered, opaque envelops according to the allocation sequence. Allocation concealment was undertaken by an independent researcher. Also, the processes of enrolling the participants and administering drugs were done by two residents of dermatology. None of them had any involvement in the trial. In one group (32 patients), fluconazole 300 mg *per week* for four weeks was applied and in another group (32 patients), terbinafine 250 mg *per day* received. Four patients discontinued study and were lost to follow-up. Patients were evaluated four weeks after initiation of treatment and two weeks after treatment and scores were noted.

Laboratory tests regarding side effects were requested for all patients at each visiting time.

### 2.5. Statistical Analysis

Data analysis is provided by SPSS software version 16. All data were statistically analyzed by means of independent *t*-test: Tukey-Kramer test, Mann-Whitney, Wilcoxon test, and repeated measure analysis of variance (ANOVA). Multivariate regression models were conducted. Statistical significance was set at *P* < 0.05.

## 3. Results

In this study, 60 patients were evaluated, 32 in the terbinafine and 28 in the fluconazole group.

On the basis of Kolmogorov-Smirnov test, the SDASI changes showed normal distribution.

Table 1 shows the comparison of SDASI in three phases of study. By Tukey-Kramer test, the SDASI changes were significant during the periods between before and after treatment as well as before treatment and two weeks after treatment in both drugs (*P* < 0.001), while there were no significant changes between after treatment and two weeks after treatment.

**Figure 1:**
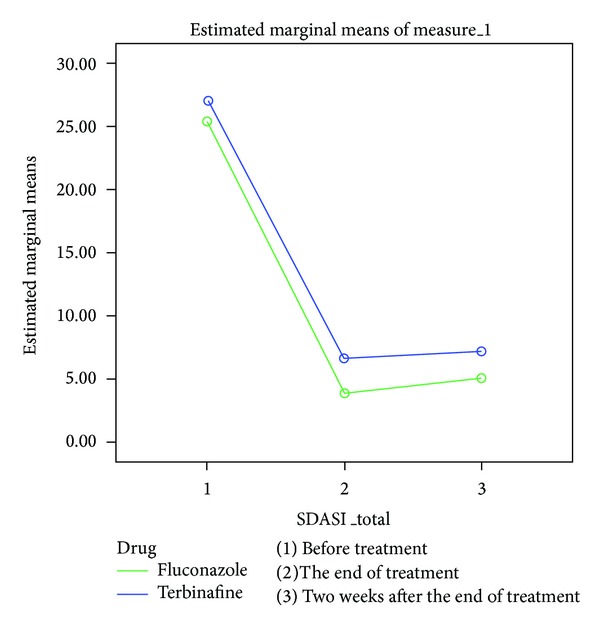
The reduction of SDASI parameters in two drugs.

By repeated measure analysis, the trend of changes were followed similarly in both drugs and there was a significant downward trend (*P* < 0.001) (Figure 1).

Independent *t*-test is applied for comparison of SDASI between two drugs in each phase; there were no significant differences between two drugs (Table 2).

By using multivariate linear regression, the mean decrease of SDASI in terbinafine group was 2.7 indexes more than fluconazole (*P* < 0.01, 95% CI (0.63–4.7)). Moreover, each index of SD severity reduced 0.9 times after treatment (*P* < 0.002, 95% CI (0.8–1.02)) (Table 3).

Table 4 shows that itching was resolved by two drugs and Mann-Whitney test revealed both drugs had similar effect in each phase.

**Figure 2:**
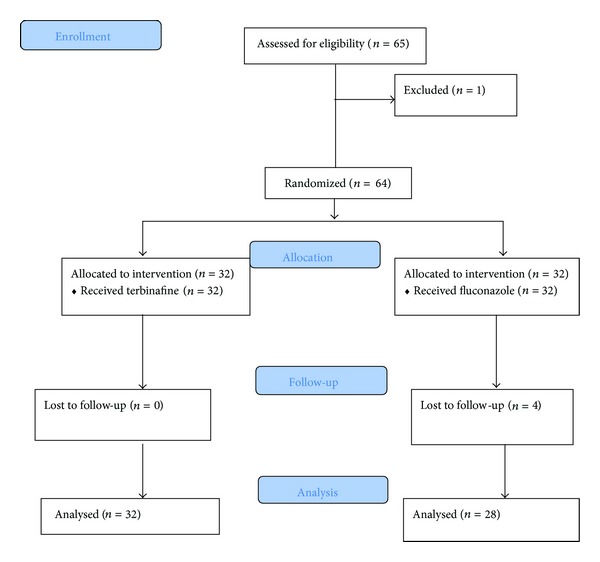
CONSORT 2010 flow diagram.

We exert Wilcoxon test to figure out the effect of drugs on itching during study and there was a significant decrease between before and after treatment in fluconazole (*P* < 0.001) and terbinafine group (*P* < 0.001) as well as between before treatment and two weeks after treatment in both drugs (*P* < 0.001). In comparison to fluconazole, terbinafine showed persistent effect on resolving itching after treatment (*P* < 0.008). There were no adverse drug effects. Flow diagram of this study is shown in Figure 2.

## 4. Discussion

In this study, both drugs (terbinafine and fluconazole) were effective as an independent treatment, while terbinafine reduced SDASI more than fluconazole. Also the severity of the disease or higher indexes was another determinant. It means that SD cases with higher index responded better during drug therapy and one SDASI may decrease 0.9 times in the end of treatment.

Both drugs are antifungal and fluconazole has broad spectrum antifungal activity while terbinafine is not only antifungal but also anti-inflammatory with high lipophilic properties which can be distributed into sebum. Regarding response, contribution of multiple factors rather than fungi in seborrheic dermatitis was inferred, though we eliminated seasonal impact. There are few studies regarding comparison of efficacy of systemic antifungal agents in seborrheic dermatitis. However different studies emphasize that one-month systemic therapy could delay recurrence and resolving persistent disease [5–8, 10]. It's better to point that we prescribed fluconazole with higher dose to reach the optimal minimal inhibitory concentration (MIC) [11]. It was showed that the maximum maintenance effect of drugs was for two weeks after treatment with a slightly increasing SDASI after this time, while itching was more plausible to improve without recurrence; however, terbinafine was more effective. Though findings revealed that fluconazole had advantages in decreasing severity of seborrheic dermatitis, consuming once every week is more pleasant for patients. We have to keep in mind that terbinafine and fluconazole could not resolve disease completely, but both reduce indexes.

Fortunately there were not any adverse effect during the period of study; however, some authors reported adverse reactions by therapy [2, 8].

Based on cultural factors, we considered short follow-up period as one of the limitations of this study. Also, the assessment of itching was subjective and based on patients' opinions.

Totally, although there is no definite guideline to treat moderate to severe seborrheic dermatitis, oral antifungal agents with anti-inflammatory effect may have reasonable response to disease.

## Figures and Tables

**Table 1:** Comparison of mean SDASI in three phases study.

Drugs	Before treatment	After treatment	Two weeks after treatment
	Mean SDASI ± SD	Mean SDASI* ± SD	Mean SDASI* ± SD
Fluconazole	27.07 ± 11.20	6.64 ± 5.05^†^	7.17 ± 3.51^†^
Terbinafine	25.64 ± 8.2	3.81 ± 2.81^†^	5.05 ± 3.83^†^

SDASI: seborrheic dermatitis area severity index.

^†^ *P* < 0.001 (Tukey-kramer *t*-test).

**Table 2:** The effects of terbinafine and fluconazole on decreasing of SDASI.

Mean decrease of SDASI	Fluconazole	Terbinafine	*P* value*
	Mean ± SD	Mean ± SD	
Before and after treatment	20.42 ± 12.34	21.65 ± 6.58	0.64
After treatment and two weeks after treatment	−0.53 ± 4.5	−1.25 ± 2.89	0.48
Before and two weeks after treatment	19.89 ± 10.51	20.4 ± 6.34	0.82

*Independent *t*-test.

**Table 3:** The effects of primary SDASI and drugs on decreasing of SDASI.

	Unstandardized coefficients		*P* value	95% confidence interval for *β*	
	*B*	Std. error		Lower bound	Upper bound
(Constant)	−6.987	2.256	0.003	−11.504	−2.471
Primary SDASI	0.913	0.054	0.000	0.806	1.021
Terbinafine versus fluconazole	2.691	1.029	0.011	0.632	4.751

**Table 4:** Comparison of itching in three-phase study.

	Drugs	*N* (%)	*N* (%)	*N* (%)	*N* (%)	Mean itching ± SD	P*
		Without itching	Mild itching	Moderate itching	Severe itching		
Before treatment	Fluconazole (28)	0 (0%)	15 (53.6%)	6 (21.4%)	7 (25%)	1.71 ± 0.85	0.459
	Terbinafine (32)	4 (12.5%)	7 (21.9%)	11 (34.4%)	10 (31.2%)	1.84 ± 1.019	

After treatment	Fluconazole (28)	15 (53.6%)	10 (35.7%)	3 (10.7%)	0 (0%)	0.57 ± 0.69	0.220
	Terbinafine (32)	15 (46.9%)	6 (18.8%)	11 (34.4%)	0 (0%)	0. 87 ± 0.90	

Two weeks after treatment	Fluconazole (28)	16 (57.1%)	9 (32.1%)	3 (10.7%)	0 (0%)	0. 53 ± 0.69	0.773
	Terbinafine (32)	18 (56.2%)	14 (43.8%)	0 (0%)	0 (0%)	0. 43 ± 0.50	

*Mann-Whitney test.
